# Conservation properties of non-conforming embedded finite-element methods based on lagrange multipliers

**DOI:** 10.1007/s10543-025-01075-8

**Published:** 2025-07-18

**Authors:** Maria Giuseppina Chiara Nestola, Patrick Zulian, Marco Favino, Rolf Krause

**Affiliations:** 1 Euler institute, Faculty of Informatics, Via La Santa 1, Viganello, 6962 Switzerland; 2 Faculty of Mathematics and Computer Science, UniDistance Suisse, Brig-Glis, 3900 Switzerland; 3 AMCS, Faculty of Computer, Electrical and Mathematical Science and Engineering, King Abdullah University of Science And Technology, Thuwal, 23955 Saudi Arabia

**Keywords:** Discrete fracture model, Flow in fractured porous media, Finite-element method, Non-conforming meshes, Variational transfer, Lagrange multiplier method, 35-XX, 65N30

## Abstract

Numerical simulations of Darcy flow in fractured porous media rely on hybrid- or equi-dimensional fracture models. The former considers fractures as lower-dimensional manifolds, while the latter treats them as objects of the same geometrical dimension as the porous matrix. Embedded strategies remove the inherent difficulties in mesh generation for fractured media, as they employ two different non-conforming meshes. While the Continuous Galerkin discretization has been shown to be locally conservative, this property has yet to be investigated for embedded strategies. This paper demonstrates that embedded strategies, based on dual Lagrange multiplier and discretized within a Continuous Galerkin framework, are locally conservative. We conduct a numerical analysis of the conservation properties in both hybrid- and equi-dimensional models for fractured porous media. Our results strongly support the conservation properties of embedded strategies.

## Introduction

Numerical simulations of flow in fractured porous media are crucial in many geophysical applications, such as geothermal energy extraction, CO$_2$ sequestration, nuclear waste storage, and unconventional oil and gas recovery [1, 4, 5, 25, 32, 39].

Fractures are heterogeneities within a surrounding background matrix, characterized by a dimension, typically referred to as aperture, which is significantly smaller than the others, and by material properties that are usually extremely different from those of the background matrix. Fractures can be arranged in complex networks, and heavily affect the flow dynamics depending on their material properties. Hence, modeling fluid flow in fractured porous media can be viewed as a diffusion problem characterized by heterogeneous and potentially anisotropic permeability that varies between the background matrix and the fractures.

One of the primary challenges in simulating fractured media using heterogeneous diffusion models is the generation of a suitable mesh. Creating meshes that resolve the numerous interfaces between the fractures and the surrounding background matrix is a complex, time-consuming task that is difficult to automate fully. Additionally, even when such meshes can be generated, they often feature elongated elements that can significantly affect the accuracy of the numerical solutions or the computational cost.

Due to these limitations, alternative modeling approaches are commonly employed. One of the primary alternatives is the use of embedded models, which treat the surrounding background matrix and the fracture network as separate entities, while enforcing the continuity, often through the use of Lagrange multipliers [2, 11]. Embedded models are commonly employed either in hybrid-dimensional settings, where fractures are objects with lower geometrical dimensions, or in equi-dimensional settings, where fracture thickness is explicitly represented. Hence, embedded models utilize two distinct meshes, which are typically not conforming. This significantly simplifies the mesh generation process by allowing independent representations of the background matrix and the fracture network. On the other hand, embedded models require suitable coupling conditions for the equations defined on the two meshes.

Mortar methods are widely used across various fields to couple different discretization schemes. These methods introduce a mortar variable (Lagrange multiplier), which can be defined either at the interface between the fracture and the background matrix or across the entire overlapping region. In [19], the authors present an approach based on a Lagrange multiplier to couple the fracture network and the surrounding background matrix. Similarly, in [36, 45], the authors highlight the advantages of the dual-Lagrange multiplier approach [31, 40], demonstrating its positive impact on the condition number, the practical performance, and the applicability of solution methods with optimal complexity (e.g., Multigrid methods).

For the discretization of models based on embedded strategies, various schemes have been developed, including finite-volume (FV) [16, 23, 28], finite-difference (FD) [26, 27], continuous Galerkin (CG) and discontinuous Galerkin (DG) finite-element methods, as well as extended finite-element approaches [7, 37, 38, 41, 42]. Each scheme offers different characteristics in terms of accuracy, stability, and mass conservation, with the latter being the focus of this manuscript. Mass conservation is a fundamental principle in the modeling of fluid flow. The continuity equation mathematically expresses the principle of mass conservation: the temporal rate of change of mass within a control volume equals the net mass flux through its boundary. This principle underpins various numerical methods used in simulating fluid flows, ensuring accurate predictions of flow behavior.

FV method is well-known to ensure local conservation properties by construction, whereas conservation properties for the FE method hold naturally for mixed formulations based on Raviart-Thomas and hybrid high order discretizations [6, 8, 9, 14, 24, 33], or discontinuous Galerkin (DG) methods [10, 34], but they can also be proven for enriched continuous Galerkin methods [22, 38], X-FEM [12, 13, 35], and the embedded finite-element methods (EFEM) [29].

CG methods are traditionally considered to be non-conservative. However, the authors in [17] established global and local conservation laws for CG discretization by a suitable choice of the unitary test function on subdomains consisting of a union of elements. Specifically, they demonstrated that:

1. global conservation is achieved by using an equivalent formulation of the problem that incorporates an auxiliary field, which represents the flux on the Dirichlet boundary;
2. local conservation is guaranteed when the equivalent formulation of the problem includes the auxiliary boundary flux for the specific subdomain being analyzed.

Modeling flow in fractured porous media as a diffusion problem with heterogeneous properties does not necessitate specialized numerical approaches. Therefore, mass conservation depends on the discretization framework adopted.

As mentioned before, embedded discretizations require specific coupling strategies. Hence, it must be demonstrated that the resulting numerical approach is both globally and locally conservative for subdomains composed of connected elements. Additionally, point-wise flux balance should be achieved to ensure uniqueness at the interfaces between subdomains. To this end, we extend the results proposed by Hughes [17] to embedded non-conforming strategies for flow in fractured porous media. Specifically, we investigate the conservative structure of embedded non-conforming finite element (FE) methods with dual Lagrange multipliers, as validated in [2, 45]. We adapt Hughes’ framework to non-conforming models discretized using a continuous Galerkin (CG) approach.

Our approach introduces an auxiliary boundary flux field for both the background matrix and the fracture subdomain and employs the coupling condition to guarantee pressure continuity. The use of dual Lagrange multipliers is critical, as it enables static condensation and facilitates the computation of nodal fluxes that account for contributions from both the matrix and fractures. To establish uniqueness, we compute point-wise fluxes and compare them to numerical results obtained using an equi-dimensional CG framework, where fracture heterogeneity is captured by varying the material parameters of the diffusion problem. The main contributions of our paper are:

1. establishing local and pointwise flux conservation for both hybrid-dimensional and equi-dimensional approaches;
2. demonstrating that the pointwise distribution of the physical flux can only be accurately recovered using equi-dimensional approaches. This is because hybrid-dimensional strategies represent fracture networks as lower-dimensional objects, which inherently limits their ability to capture the flux discontinuity across the fracture aperture.

This paper is organized as follows. In Sect. 2 we summarize the main features of the mathematical model presented in  [45]. In Sect. 3 we introduce the definition of flux conservation by generalizing the Hughes approach to the strategy described in Sect. 2, whereas all the numerical results are presented in Sect. 4. Conclusions are presented in Sect. 5. In the appendix 6 we provide some numerical computations extending Sect. 3.

## Embedded strategies for flow in fractured porous media

We formulate the coupled flow problem for the surrounding background and the fracture network by using an embedded strategy based on the method of dual Lagrange multipliers. Fractures are represented as a separate body embedded in the background matrix and are described by either a lower-dimensional manifold or an equi-dimensional domain. This choice allows us to flexibly deal with mesh non-conformity and employ $L^2$–projections  [20] to couple the background matrix and fracture problems.

**Fig. 1:**
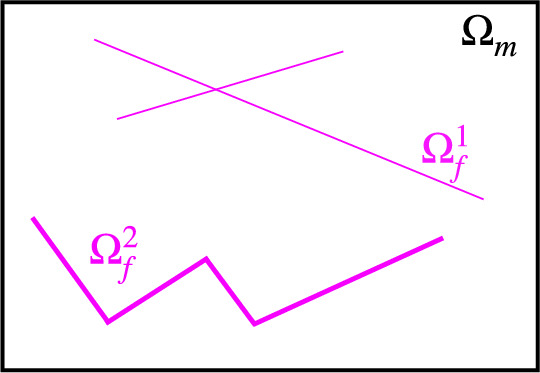
Two-dimensional $d=2$ representation of a background matrix where a hybrid dimensional ($\Omega _f^{d-1}=\Omega _f^1$) and an equi-dimensional fracture ($\Omega _f^d=\Omega _f^2$) are embedded

**Fig. 2:**
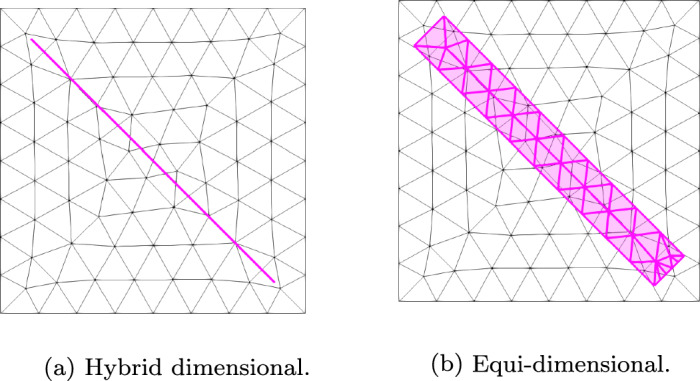
Embedded non-matching mesh representations of background matrix (black) and fracture (thick/purple). Hybrid-dimensional (left) and equi-dimensional (right) fractures

Figure 1 provides an overview of the different continuous geometric representations of both the background matrix and the fractures. The associated symbols and their definitions are detailed in Section 2.1, while Fig. 2 illustrates a hybrid-dimensional and an equi-dimensional mesh representing a fracture embedded in the mesh of the background matrix.

We point out that the method has been numerically validated and verified by a systematic comparison against other discretization approaches in  [2, 45]. Thus, this paper does not aim at presenting a novel methodology but focuses on analyzing the conservation properties of the numerical approach presented in [45]. For the sake of completeness, we present a small overview of the main features of the mathematical model, which are essential for the purpose of this paper. However, we recommend the reader to refer to [45] for a more detailed and complete description of the embedded strategy and its discretization.

### Preliminary definition

We define $\Omega _m \subset \mathbb {R}^d$, with $d \in \{2, 3\}$, as the surrounding background. We also introduce $\Omega _f^{\tilde{d}} \subset \Omega _m$ as a domain of dimension $\tilde{d}=\{d,d-1\}$ describing either a lower dimensional ($\Omega _f^{d-1}$) or an equi-dimensional ($\Omega _f^d$) fracture network.

In the following section, we use the subscript $a\in \{m,f\}$, where *m* refers to the background matrix and *f* refers to the fracture network. Additionally, we omit the superscript $\tilde{d}$ for ease of notation.

The function space $V_a$ with $a\in \{m,f\}$ is defined by:

$$\begin{aligned} V_a = H_0^1(\Omega _a)\,\,\text {with}\,\,a\in \{m,f\}, \end{aligned}$$

where $H^1_0 \subset H^1$ is its restriction to functions vanishing at the boundary. We denote $H^{-\frac{1}{2}}_{00}(\Omega ^{d-1}_{f})$ and $H^{-1}_{00}(\Omega _{f}^{d})$ as the dual spaces of $H^{\frac{1}{2}}(\Omega ^{d-1}_{f})$ and $H^{1}(\Omega ^{d}_{f})$ with zero-extension outside $\Omega _f$, respectively [15]. The Lagrange multiplier space $\Lambda (\Omega _f)$ is defined as:

$$ \Lambda = H^{-\frac{1}{2}}_{00}(\Omega _f^{d-1}) $$

for the hybrid-dimensional models and as

$$ \Lambda = H^{-1}_{00}(\Omega _f^{d}) $$

for the equi-dimensional models.

We denote the inner product associated with the function space $L^2(D)$ as $(\cdot , \cdot )_{D}$. The boundary of each domain $\Omega _a$ is denoted as $\Gamma _a$ and split into a Dirichlet $\Gamma ^D_a$ and a Neumann $\Gamma ^N_a$ portion with $a\in \{m,f\}$, such that $\Gamma ^N_a\cap \Gamma ^D_a=\emptyset$.

### Flow problem

The Darcy fluid flow in the background matrix $\Omega _m$ is governed by the following boundary-value problem:

2.1 $$\begin{aligned} \begin{array}{c c c l} \nabla \cdot (-\varvec{K}_m\nabla p_m) & = &  0 &  \quad \text {in } \Omega _m,\\ \varvec{K}_m\nabla p_m \cdot \varvec{n}&  = &  f_m &  \quad \text {on} \,\, \Gamma _m^N, \\ p_m &  = &  0, &  \quad \text {on} \,\, \Gamma _m^D, \end{array} \end{aligned}$$

where $p_m$ is the pressure, $f_m$ a prescribed flux, and $\varvec{K}_m \in \mathbb {R}^{d \times d}$ a permeability tensor, which is usually assumed to be symmetric and positive definite. However, for the purpose of this work and without loss of generality, we can assume that $\varvec{K}_m=\textbf{I}$, with $\textbf{I}$ being the identity tensor.

Flow in the fracture network $\Omega _f$ is described by the boundary-value problem:

2.2 $$\begin{aligned} \begin{array}{c c c l} \nabla \cdot (-\varvec{K}_f\nabla p_f) & = &  0 &  \quad \text {in } \Omega _f,\\ p_f &  = &  0, &  \quad \text {on} \,\, \Gamma _f^D, \end{array} \end{aligned}$$

where $p_f$ is the pressure, $\varvec{K}_f$ is a given permeability symmetric positive definite tensor. We assume that homogeneous Neumann boundary conditions are applied on $\Gamma _f\smallsetminus \Gamma _f^D$. The lower-dimensional fracture is associated with a thickness parameter, i.e., the fracture aperture $\varepsilon >0$, which represents a uniform scaling factor in the permeability tensor defined by: $\varvec{K}_{{f}} = \varepsilon \kappa \varvec{I}$, where $\kappa >0$ is the permeability parameter associated with the subdomain $\Omega _{f}$.

Equations (2.1) and (2.2) are coupled by means of a coupling condition prescribing the continuity of the pressure field over the overlapping region $\Omega _m\cap \Omega _f$:

2.3 $$\begin{aligned} p_f = p_m, \quad \text {on} \,\, \Omega _m\cap \Omega _f. \end{aligned}$$

The weak formulation of the coupled problem defined by Equations (2.1)-(2.3) is based on the mortar strategy previously introduced in [45] and reads as follows:

2.4 $$\begin{aligned} \text {find }p_m\in V_m, \, p_f \in V_f\text { and }\lambda \in \Lambda \text {, such that}:\nonumber \\ (\nabla p_m, \nabla v_m)_{\Omega _m} - (\lambda , v_m)_{\Omega _f} =&(f_m, v_m)_{\Gamma _m^N},\, \forall \,v_m\in V_m \end{aligned}$$

2.5 $$\begin{aligned} (\nabla p_f, \nabla v_f)_{\Omega _f} +(\lambda , v_f)_{\Omega _f} =&0,\quad \quad \quad \quad \quad \forall \, v_f \in V_f \end{aligned}$$

2.6 $$\begin{aligned} (p_f - p_m, \mu )_{\Omega _f}=&0, \qquad \qquad \quad \forall \,\mu \in \Lambda , \end{aligned}$$

where the Lagrange multiplier $\lambda$ represents the flux exchange between $\Omega _m$ and $\Omega _f$. We point out that since we only refer to the case in which fracture networks are completely embedded in the background matrix domain, $\Omega _f$ represents the overlapping region, i.e. $\Omega _f=\Omega _m\cap \Omega _f$.

### Finite element discretization

Let $\mathcal {T}_a = \mathcal {T}_{\Omega _a}$ with $a \in \{m, f\}$ be regular partitions of polytypic domains of background matrix and fracture-network, respectively.

For manifolds with dimension $\tilde{d}$ with Lagrange elements $\mathbb {P}^k$, or tensor-product elements $\mathbb {Q}^{k}$ of order *k*, the finite element spaces of the pressure fields are

$$\begin{aligned} \begin{aligned} V_a^h = \{&v_a \in C^0(\mathcal {T}_a) \, | \, v_a=0 \, \text {on} \, \Gamma _a^D :\,\forall E \in \mathcal {T}_a \\&v_a |_{E} \in \left\{ \begin{aligned} \mathbb {P}^k&\quad \text {if}~E~\text {is a simplex} \\ \mathbb {Q}^{k}&\quad \text {if}~E~\text {is a hyper-cuboid} \end{aligned} \right\} \\ \}, \\&a \in \{ m, f\}. \end{aligned} \end{aligned}$$

With $\Lambda _h = \Lambda _h(\mathcal {T}_f)$ we denote the finite element space of dual Lagrange multipliers [31, 40, 45]. Let $\{ \varphi ^i_a \}_{i \in \mathcal {N}_a}$ be a basis of $V_a^h$, and $\{ \psi ^k_f \}_{k \in \mathcal {N}_f}$ a basis of $\Lambda _h$, where $\mathcal {N}_a \subset \mathbb {N}$ are index sets of the node-sets of their respective meshes $\mathcal {T}_a$, $a \in \{m, f\}$. Writing the functions $v_a \in V_a^h$, and $\mu \in \Lambda _{h}$ in terms of their respective bases and coefficients, they read $v_a = \sum _{i \in \mathcal {N}_a} v_a^i \varphi _a^i$, and $\mu = \sum _{k \in \mathcal {N}_f} \mu ^{k} \psi _f^k$.

The dual shape functions $\psi _f^k \in \Lambda _h$ are constructed to satisfy the bi-orthogonality condition [40]:

$$\begin{aligned} (\varphi ^i_f, \psi _f^j)_{\Omega _f} = \delta _{ij} (\varphi ^i_f, 1)_{\Omega _f} \qquad \forall i,j \in \mathcal {N}_{f}, \end{aligned}$$

where $\delta _{ij}$ is the Kronecker delta function and integral positivity

2.7 $$\begin{aligned} (\psi _f^j, 1)_{\Omega _f} > 0. \end{aligned}$$

Note that (2.7) is naturally satisfied for first-order finite elements. For second-order elements, we follow the construction described in [21, 31].

The variational problem (2.4)–(2.6) is expressed as a set of point-wise algebraic equations. The discrete problem for the porous background matrix (2.4) reads:

2.8 $$\begin{aligned} \sum _{i \in \mathcal {N}} p_m^i ( \nabla \varphi _m^i, \nabla \varphi _m^j)_{\Omega _m} - \sum _{k \in \mathcal {N}_f} \lambda ^k (\psi _f^k, \varphi _f^j)_{\Omega _f} = (f_m, \varphi _m^j)_{\Omega _n} \qquad \forall j \in \mathcal {N}_m, \end{aligned}$$

which translates to the linear system $\textbf{A}_m \textbf{p}_m - \textbf{B}^T \varvec{\lambda } = \textbf{f}_m$. The fracture sub-problem (2.5) results in,

2.9 $$\begin{aligned} \sum _{i \in \mathcal {N}_f} p_f^i (\nabla \varphi _m^i, \nabla \varphi _m^j)_{\Omega _f} + \sum _{k \in \mathcal {N}_f} \lambda ^k (\psi _f^k, \varphi _f^j)_{\Omega _f} = 0 \qquad \forall j \in \mathcal {N}_f, \end{aligned}$$

which translates to the linear system $\textbf{A}_f\textbf{p}_f + \textbf{D}^T \varvec{\lambda } = \textbf{0}$. The weak-equality condition (2.6) results in,

2.10 $$\begin{aligned} - \left( \sum _{i \in \mathcal {N}_f} p_m^i (\varphi _m^i, \psi _f^j)_{\Omega _f} - \sum _{k \in \mathcal {N}_f} p_{f}^k (\mathbf \varphi _f^k, \psi _f^j)_{\Omega _f} \right) = 0 \qquad \forall j \in \mathcal {N}_f, \end{aligned}$$

which translates to the linear system $-\textbf{B} \textbf{p}_m + \textbf{D} \textbf{p}_f = \textbf{0}$. It is important to note that all the integrals involving the evaluation of the Lagrange multipliers and their basis functions necessitate the use of numerical quadrature procedures based on intersection meshes [20].

The overall saddle-point system including Equations (2.8)–(2.10) is

$$\begin{aligned} \left| \begin{array}{ccc} \textbf{A}_m &  \textbf{0} &  -\textbf{B}^T \\ \textbf{0} &  \textbf{A}_f &  \textbf{D}^T \\ -\textbf{B} &  \textbf{D} &  \textbf{0} \end{array} \right| \left| \begin{array}{l} \textbf{p}_m \\ \textbf{p}_f\\ \varvec{\lambda } \end{array} \right| = \left| \begin{array}{l} \textbf{f}_m \\ \textbf{0} \\ \textbf{0} \end{array} \right| . \end{aligned}$$

Due to the choice of dual Lagrange multipliers, the mass matrix $\textbf{D}$ can be inverted trivially. Indeed, for linear elements, the mass matrix $\textbf{D}$ is diagonal, whereas for quadratic elements, the inverse is constructed as a combination of a basis transformation, for which its inverse is known [31]. This convenient property enables us to perform static condensation and obtain the following system [30]

$$\begin{aligned} (\textbf{A}_m + \textbf{T}^T \textbf{A}_f \textbf{T}) \textbf{p}_m = \textbf{f}_m, \end{aligned}$$

where $\textbf{T} = \textbf{D}^{-1}\textbf{B}$. Once this system is solved for $\textbf{p}_m$, the solution for the fracture network can be computed by $\textbf{p}_f = \textbf{T}\textbf{p}_m$. Further details about this technique are found in [36, 45].

## Conservation properties

In this section, we analyze the conservative properties of coupled embedded strategy based on dual Lagrangian multipliers. In Subsection 3.2, we consider a case for which no fractures are embedded in the surrounding background and examine the conservation properties of the CG method. In Subsection 3.3, we consider the general case where a fracture network is embedded in the background matrix and explore the conservative structure of the embedded strategies introduced in Sect. 2.

### Preliminary definitions

We let $D_a$ be the subset of the nodes located on the Dirichlet boundary

$$D_a = \{ i \,: \, \textbf{x}_i \in \bar{\Gamma }_a^D\},$$

and introduce the following function space

$$\begin{aligned} {S}^h_a = {V}^h_a \bigoplus \textrm{span}\{\varphi _a^i\}_{i \in D_a}\,\,\,\text {with}\,a\in \{m,f\}, \end{aligned}$$

where $\textrm{span}\{\varphi _a^i\}_{i \in D_a}$ is the span of the basis functions associated with the set of nodes in $D_a$. Note that ${S}^h_a,\text {with}\,a\in \{m,f\}$ contains the unit constant functions over $\Omega _a$.

**Fig. 3:**
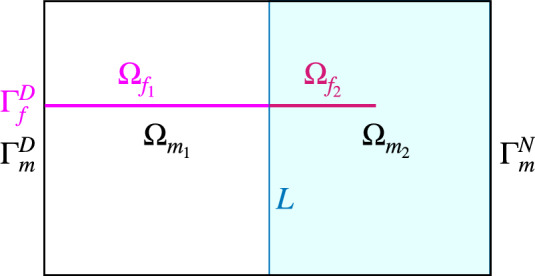
Geometric set-up for the study on local conservation properties. $\Gamma _{f}^D$ is the Dirichlet boundary of fracture domain $\bar{\Omega }_{f} = \bar{\Omega }_{f_1} \cup \bar{\Omega }_{f_2}$. $\Gamma _m ^D$ and $\Gamma _m ^N$ denote the Dirichlet and Neumann boundaries of the background matrix $\bar{\Omega }_{m} = \bar{\Omega }_{m_1} \cup \bar{\Omega }_{m_2}$

Figure 3 depicts a single fracture embedded in the background matrix and intersecting the Dirichlet boundary. Note that, the proposed analysis remains valid for internal fractures entirely enclosed within the domain. The conservation statement extends naturally to complex scenarios involving arbitrary fracture networks embedded within a three-dimensional background matrix, as demonstrated in Sect. 4.

We define $\Omega _{m_1} \subset \Omega _m$ and $\Omega _{m_2} \subset \Omega _m$ as two subdomains such that $\bar{\Omega }_m=\bar{\Omega }_{m_1}\cup \bar{\Omega }_{m_2}$ and ${\Omega }_{m_1}\cap {\Omega }_{m_2}=\emptyset$. We denote the boundary of the subdomain $\Omega _{m_k}$ by $\Gamma _{m_k}$ with $k\in \{1,2\}$, and $\bar{L}=\bar{\Omega }_{m_1}\cap \bar{\Omega }_{m_2}$ as the interface along which we evaluate the conservation properties in the background matrix. Moreover, we assume that $\Gamma _{D}\subset \Gamma _{m_1}{\setminus }L$ and $\Gamma _{m}^{N}\subset \Gamma _{m_2}{\setminus }L$. It is worth pointing out that the additional line *L* is not part of either the matrix or the fracture mesh; it is introduced exclusively for post-processing purposes.

We define an interface into the fracture network as $\bar{l}=L\cap {\bar{\Omega }_f}$, which induces a domain decomposition such that $\Omega _{f_k}=\Omega _{m_k}\cap \bar{\Omega }_f$ with $k\in \{1,2\}$. The interface *l* is a subset of $\bar{\Omega }_f$, which can also be an empty set.

Moreover, we refer to $J_m$ as the set of nodes residing on $\bar{L}$ and to $J_f$ as the set of nodes residing on $\bar{l}$, and denote

$$G_{a}^h = \textrm{span} \{ \varphi _a^i \}_{\{i \in J_a \}} \text {with}\,a\in \{m,f\}.$$

To derive the local conservation properties, we denote the approximated interface flux with respect to ${\Omega }_{a_k}$ as $q({{\Omega }_{a_k}})$ with $a\in \{m,f\}$ and $k\in \{1,2\}$.

Table 1 summarizes the main notations used in Subsections 3.2 and 3.3.

**Table 1:** Fundamental notations. Here $a\in \{m,f\}$ and $k=\{1,2\}$

Symbol	Definition
$\Omega _{a_k}$	Subdomain of $\Omega _{a}$
$\bar{L}$	$\bar{\Omega }_{m_1}\cap \bar{\Omega }_{m_2}$
$\bar{l}$	$l=L\cap \bar{\Omega }_f$
$q({\Omega }_{a_k})$	Interface flux with respect to $\Omega _{a_k}$
$\textbf{q}({{\Omega }_{a_k}})$	Coefficient vector of the nodal values of the flux $q({\Omega }_{a_k})$
$\textbf{q}_k$	Coefficient vector of the nodal values of the interface flux including
	contributions from the background matrix and the fracture sub-problems
$\textbf{M}_L$	Boundary mass matrix on interface *L*
$\textbf{M}_l$	Boundary mass matrix on interface *l*
$\textbf{M}_a^D$	Boundary mass matrix on the Dirichlet boundary $\Gamma _a^D$
$J_m$	Set of nodes residing on $\bar{L}$
$J_f$	Set of nodes residing on $\bar{l}$

### Local conservation properties of the CG method

We first summarize the main idea behind the proof of the local conservation presented in [17]. We refer to a diffusion problem, such as the flow problem  (2.1) defined on a domain $\Omega _m$ without any fracture. Thus, we examine the local conservation properties of the following CG formulation:

3.1 $$\begin{aligned} \begin{array}{l} \text {find } p_m\in V_m^h \, \text { such that }\\ {\displaystyle (\nabla p_m, \nabla v_m)_{{\Omega }_{m}} - ( f_m , v_m)_{\Gamma _m^N} =\,0 \, \quad \forall v_m \in V_m^h,} \end{array} \end{aligned}$$

where we omit the superscript *h* for all the discrete functions for the sake of simplicity.

The interface flux $q({\Omega }_{m_1})$ associated with the subdomain ${\Omega }_{m_1}$ and computed across the interface *L* is the solution of the following formulation:

3.2 $$\begin{aligned} \begin{array}{l} \text {find } p_m \,\in V_m^h, \,\text {and} \, q({\Omega }_{m_1}) \in G_{m}^h \,\, \text { such that }\\ (q({\Omega }_{m_1}),w_m)_{L}=(\nabla p_m , \nabla w_m)_{{\Omega }_{m_1}} - (q_D({\Omega }_{m}), w_m)_{\Gamma _m^D} \quad \forall w_m\in S_m^h, \end{array} \end{aligned}$$

where $(q_D({\Omega }_m),w_m)_{\Gamma _m^D}$ is the boundary flux associated with the Dirichlet boundary condition defined on $\Gamma _m^D$, and $w_m$ are the test functions associated with the function space $S_m^h$.

In the same way, one can define the interface flux for the complementary domain $q({\Omega }_{m_2})$ across the interface *L* as the solution to the problem:

3.3 $$\begin{aligned} \begin{array}{l} \text {find }p_m\in V_m^h, \, \text {and} \,\, q({\Omega }_{m_2})\,\in G_{m}^h \, \text {such that }\\ (q({\Omega }_{m_2}),w_m)_{L}=(\nabla p_m , \nabla w_m)_{{\Omega }_{m_2}} - (f_m, w_m)_{\Gamma _m^N} \quad \forall \, w_m \in S_m^h. \end{array} \end{aligned}$$

We establish the local conservation by first proving that the interface fluxes, $q({\Omega }_{m_1})$ and $q({\Omega }_{m_2})$, are conservative with respect to the corresponding subdomains ${\Omega }_{m_1}$ and ${\Omega }_{m_2}$.

The conservation laws implied by Equations (3.2) and (3.3) are established by selecting any $w_m\in S^h_m$ such that ${w}|_{{\Omega }_{m_k}}=1$ with $k\in \{1,2\}$. With this selection, we get

$$\begin{aligned} (q({\Omega }_{m_1}),1)_{L} + (q_D({\Omega }_{m}),1)_{\Gamma _m^D} =0, \end{aligned}$$

and

$$\begin{aligned} (q({\Omega }_{m_2}),1)_{L}+ (f_m,1)_{{\Gamma }_m^N}=0. \end{aligned}$$

It only remains to prove the uniqueness of the interface flux over *L*.

Summing together Equation (3.2) and Equation (3.3) and restricting *w* to $G_{m}^h\subset S^h_m$ leads to

3.4 $$\begin{aligned} (q({\Omega }_{m_1})+ q({\Omega }_{m_2}),w_m)_{L}=(\nabla p_m, \nabla w_m)_{{\Omega }_m} - ( f_m , w_m)_{\Gamma _m^N}=0, \end{aligned}$$

which is equivalent to the problem

$$ \sum _{i\in J_m}(\varphi _m^j,\varphi _m^i)_L(q^i({\Omega }_{m_1})+q^i({\Omega }_{m_2}))=0\quad \forall \, j\in J_m, $$

where $q^i({\Omega }_{m_k})$ are the nodal values of the interface flux $q(\Omega _{m_k})$ with $k\in \{1,2\}$.

Thus, it follows that the two interface fluxes balance each other point-wise on the interface *L*.

The algebraic representation of Equation (3.4) is,

$$\begin{aligned} \sum _{k\in \{1,2\}}\sum _{i\in J_m}(\textbf{M}_L\textbf{q}({\Omega }_{m_k}))_i=0, \end{aligned}$$

where we refer to Table 1 for the meaning of each term.

### Local conservation of embedded strategies based on the CG method

We generalize the approach described in Subsection 3.1 to the coupled problem defined by Equations  (2.4)–(2.6). Thus, we introduce the fields $q({\Omega }_{a_k}),\,\text {with} \,\,a \in \{m,f\}\,\,\text {and}\,\,k \in \{1,2\}$, defining the fluxes associated with the background matrix subdomains ${\Omega }_{m_k}$, and the fracture network subdomains ${\Omega }_{f_k}$, respectively.

The interface flux $q({\Omega }_{m_1})$ is obtained as the solution of the following problem:

3.5 $$\begin{aligned} \begin{array}{l} \text {find } p_m\in V_m^h, \lambda \in \Lambda _h,\, \text {and} \, q({\Omega }_{m_1}) \in G_{m}^h \text { such that }\\ (q(\Omega _{m_1}), w_m)_{L}= ( \nabla p_m, \nabla w_m)_{{\Omega }_{m_1}} - (\lambda , w_m)_{{\Omega }_{f_1}} -(q_D({\Omega }_{m}), w_m)_{\Gamma _m^D} \quad \forall \,w_m\in S_m^h. \end{array} \end{aligned}$$

The counterpart $q(\Omega _{m_2})$ is determined as the solution of the following problem:

3.6 $$\begin{aligned} \begin{array}{l} \text {find } p_m\in V_m^h, \lambda \in \Lambda _h,\, \text {and} \, q(\Omega _{m_2}) \in G_{m}^h \text { such that }\\ (q(\Omega _{m_2}), w_m)_{L}= (\nabla p_m, \nabla w_m)_{{\Omega }_{m_2}} - (\lambda , w_m)_{{\Omega }_{f_2}} - (f_m, w_m)_{\Gamma _m^N}\quad \forall \,w_m\in S_m^h. \end{array} \end{aligned}$$

It is worth pointing out that $(\lambda ,v_m)_{{\Omega }_{f_k}}$ with $k\in \{1,2\}$ represents the flux exchange between ${\Omega }_{m_k}$ and ${{\Omega }_{f_k}}$.

Similarly, the interface flux $q({\Omega }_{f_1})$ is computed as the solution of the following problem:

3.7 $$\begin{aligned} \begin{array}{l} \text {find} \,p_f\in V_f^h, \lambda \in \Lambda _h,\, \text {and} \, q(\Omega _{f_1}) \in G_{f}^h \text { such that } \\ (q({\Omega }_{f_1}), v_f)_{l}= (\varvec{K}_{f} \nabla p_f, \nabla w_f)_{{\Omega }_{f_1}} + (\lambda , w_f)_{{\Omega }_{f_1}} -(q_D({\Omega }_{f}), w_f)_{\Gamma _f^D} \quad \forall \,w_f\in S_f^h. \end{array} \end{aligned}$$

We note that the numerical flux associated with homogeneous Neumann boundary conditions is zero. However, the proposed formulation can be naturally extended to handle non-homogeneous Neumann conditions by incorporating the corresponding boundary flux terms into the weak formulation.

The counterpart $q({\Omega }_{f_2})$ is the solution of the following formulation:

3.8 $$\begin{aligned} \begin{array}{l} \text {find} \,p_f\in V_f^h, \lambda \in \Lambda _h,\, \text {and} \, q(\Omega _{f_2}) \in G_{f}^h \text { such that } \\ (q({\Omega }_{f_2}), w_f)_{l}= (\varvec{K}_{f} \nabla p_f, \nabla w_f)_{{\Omega }_{f_2}} + (\lambda , w_f)_{{\Omega }_{f_2}} \quad \forall \,w_f\in S_f^h. \end{array} \end{aligned}$$

Equations (3.5) and (3.7), and Equations (3.6) and (3.8) are coupled using the continuity condition

$$\begin{aligned} (p_f-p_m, \mu )_{{\Omega }_f}=0. \quad \forall \mu \in \Lambda ^h. \end{aligned}$$

By selecting any $v_a\in S^h_a$ such that ${v}|_{{\Omega }_{a_k}}=1$ with $k\in \{1,2\}$ we get

$$\begin{aligned} (q(\Omega _{a_1}),1)_{x} + (q({\Omega }_{a}),1)_{\Gamma _a^D} =0, \end{aligned}$$

and

$$\begin{aligned} (q(\Omega _{a_2}),1)_{x}+ (f_a,1)_{\Gamma _a^N}=0, \end{aligned}$$

where, $x=L$ if $a=m$, and $x=l$ if $a=f$.

By restricting $v_a$ to $G_{a}^h\subset S^h_a$ leads to

$$\begin{aligned} (q(\Omega _{a_1})+ q(\Omega _{a_2}),w_a)_{x}=0, \end{aligned}$$

which is equivalent to the following problem

$$\sum _{i\in J_a^L}(\varphi _a^j,\varphi _a^i)_{x}(q^i(\Omega _{a_1})+q^i(\Omega _{a_2}))\,\forall \, j\in J_a, \,\text {with}\, a\in \{m,f\},$$

where again $x=L$ if $a=m$, and $x=l$ if $a=f$. It follows that the two interface fluxes balance each other point-wise on the interface for both the background matrix and the fracture network sub-problems.

Equations (3.5) and  (3.6) admit the following algebraic representations

3.9 $$\begin{aligned} \textbf{M}_L\textbf{q}({\Omega _{m_1}})= \textbf{A}_{m_1}\textbf{p}_m - \textbf{B}_{1}^T \varvec{\lambda } - \textbf{M}_m^D \textbf{q}_m^D, \end{aligned}$$

and

3.10 $$\begin{aligned} \textbf{M}_L\textbf{q}({\Omega _{m_2}})= \textbf{A}_{m_2}\textbf{p}_m - \textbf{B}_{2}^T \varvec{\lambda } -\textbf{f}_m, \end{aligned}$$

where $\textbf{M}_L\textbf{q}({\Omega }_{m_k})$ is the algebraic representation of the term $(q({\Omega }_{m_k}),w_m)_{L}$, $\textbf{A}_{m_k}\textbf{p}_m$ is the algebraic representation of $( \nabla p_m, \nabla w_m)_{{\Omega }_{m_k}}$,  $\textbf{B}_{k}^T\varvec{\lambda }$ is the algebraic representation of $(\lambda , w_m)_{{\Omega }_{f_k}}$, and $\textbf{M}_m^D\textbf{q}_m^D$ is the algebraic representation of $(q_D({\Omega }_m), w_m)_{\Gamma _m^D}$.

Similarly, Equations (3.7) and (3.8) admit the following algebraic representations

3.11 $$\begin{aligned} \textbf{M}_l\textbf{q}({\Omega }_{f_1})= \textbf{A}_{{f_1}}\textbf{p}_{f} + \textbf{D}_{1}^T \varvec{\lambda }-\textbf{M}_{f}^D\textbf{q}_{f}^D, \end{aligned}$$

and

3.12 $$\begin{aligned} \textbf{M}_l\textbf{q}({\Omega }_{f_2})= \textbf{A}_{{f_2}}\textbf{p}_{f} + \textbf{D}_{2}^T \varvec{\lambda }, \end{aligned}$$

where $\textbf{M}_l\textbf{q}({\Omega }_{f_k})$ is the algebraic representation of the term $(q({\Omega }_{f_k}),w_f)_{l}$,   $\textbf{A}_{{\Omega }_{f_k}}\textbf{p}_f$ is the algebraic representation of $(\varvec{K}_f \nabla p_f, \nabla w_f)_{{\Omega }_{f_k}}$, $\textbf{D}_{k}^T\varvec{\lambda }$ is the algebraic representation of $(\lambda , w_f)_{{\Omega }_{f_k}}$, and $\textbf{M}_{f}^D\textbf{q}_{f}^D$ is the boundary flux associated with the Dirichlet boundary condition defined on $\Gamma _f^D$.

The choice of a dual Lagrange multiplier space makes $\textbf{D}$ a diagonal matrix and allows us to perform a static condensation as follows

3.13 $$\begin{aligned} \textbf{M}_L \textbf{q}_1 = \textbf{A}_{m_1}\textbf{p}_m + \textbf{T}_1^T \textbf{A}_{f_1}\textbf{T}\textbf{p}_m -\textbf{M}_m^D \textbf{q}_m^D - \textbf{T}_1^T\textbf{M}_f^D\textbf{q}^D_f , \end{aligned}$$

and

3.14 $$\begin{aligned} \textbf{M}_L \textbf{q}_2= \textbf{A}_{m_2}\textbf{p}_m + \textbf{T}_2^T \textbf{A}_{f_2}\textbf{T}\textbf{p}_m -\textbf{f}_m, \end{aligned}$$

where $\textbf{q}_k$ with $k\in \{1,2\}$ is the coefficient vector of the nodal values of the interface flux which accounts for the contribution of the background matrix and the fracture sub-problems, $\textbf{T}=\textbf{D}^{-1}\textbf{B}$, and $\textbf{T}_k=\textbf{D}_k^{-1}\textbf{B}_k$. All the algebraic manipulations needed to derive Equations (3.13) and  (3.14) are shown in the Appendix 6.

Since we have proven that local conservation is attained, then it follows that

$$\sum _{k\in \{1,2\}}\sum _{i\in J_m^L}(\textbf{M}_L\textbf{q}_k){i}=0,$$

where we refer to Table 1 for the meaning of each term. We emphasize that the analysis does not explicitly rely on properties such as bi-orthogonality or positivity of the dual Lagrange multiplier. Consequently, the same approach can also be applied to standard mortar methods. The analysis can naturally generalizes to a single element subdomain, thereby deriving the element conservation law, as demonstrated by Hughes [17].

## Numerical results

This section comprises a computational exploration of the conservation properties of FE discretizations of embedded methods based on non-conforming meshes. We apply the strategy described in the previous section to fluid flow within fractured porous media. As such, our primary focus is on assessing the conservation framework inherent in the embedded discretization strategies outlined and widely validated in [45].

As an illustrative case, we consider the two-dimensional regular fracture network benchmark proposed in [11] and the three-dimensional test case network with small features presented in [2].

We point out that the validation of the discretization for which we show the conservation properties has already been done in [2, 36, 45].

Our assessment encompasses an analysis of conservation properties of both equi-dimensional (*Embedded-ED*) and hybrid-dimensional (*Embedded-HD*) embedded approaches. Structured and unstructured meshes are used for the background matrix and the equi-dimensional fracture network.

The total scalar flux $Q_{k}$ with $k \in \{ 1,2\}$ along the interface *L* between the subdomains $\Omega _{m_1}$ and $\Omega _{m_2}$ is computed as follows:

4.1 $$\begin{aligned} Q_k=\int _L q_k\, d\Gamma = \sum _{i\in J_m}(\textbf{M}_L\textbf{q}_k)_{i}, \end{aligned}$$

where $\textbf{q}_k$ with $k\in \{1,2\}$ is the coefficient vector of the nodal values of the interface flux, as defined in Equations (3.13)-(3.14).

**Table 2:** Regular fracture network benchmark: material properties

Property	Symbol	Value	Unit
Fracture aperture	$\epsilon$	$10^{-4}$	m
Background matrix permeability	$k_m$	1	m/s
Fracture network permeability	$k_f$	$10^4$	m/s

**Table 3:** Regular fracture network benchmark: mesh characteristics for the * structured* and the *unstructured* *Embedded-HD* test cases. We report the number of refinements in the second column for each test case, the number of elements and nodes for the meshes of the background matrix (**#E-matr.**, and **#N-matr.**) and of the fracture network (**#E-frac.**, and **#N-frac.**)

**Mesh**	Test case	#E-matr.	#N-matr.	#E-frac.	#N-frac.
*Structured*	0	156	182	176	185
	1	624	675	350	359
	2	2 496	2 597	438	447
	3	9 884	10 185	583	592
	4	39 936	40 337	2 333	2 342
	5	159 744	160 545	5 600	5 609
	6	638 976	640 577	11 200	11 209
	7	2 555 904	2 559 105	23 333	23 342
*Unstructured*	0	228	135	176	185
	1	912	497	350	359
	2	3 648	1 905	438	447
	3	14 592	7 457	583	592
	4	58 368	29 505	2 333	2 342
	5	255 644	128 489	5 600	5 609
	6	1 026 360	514 515	11 200	11 209
	7	3 606 642	1 805 822	23 333	23 342

### Regular fracture network: material properties and geometrical settings

The geometry of this benchmark model features a regular fracture network embedded within a square domain with $X_0 = Y_0 = 1\,[\text {m}]$. Material properties are listed in Table 2. For the background matrix, $\Gamma _m^D$ is the right side at $x=X_0$, while $\Gamma _m^N$ is composed of the other three sides. We impose a non-homogeneous Dirichlet boundary condition, i.e., $g_m=1$ [m], on $\Gamma _m^D$ and $f_m=-1$ [m/s] on the left side of $\Gamma _m^N$, i.e., on $x=0$. On the upper and lower sides, homogeneous Neumann boundary conditions are imposed. The same boundary conditions are equivalently applied to the fracture network, as reported in [11]. In our experiments, we ensure that the meshes of both the background matrix and the fracture network satisfy the requirements shown in [18], where the authors prove that the mesh size $h_{f}$ associated with the discretization of the Lagrange multiplier (in our case directly the fracture mesh) and the mesh size of the background matrix discretization $h_{m}$, have to satisfy $h_{f}/h_{m} \ge 1$. Material parameters are summarized in Table 2 for the equi-dimensional and the hybrid-dimensional case. The fracture network of this test case has a uniform aperture, i.e $\varepsilon =10^{-4}$.

As mentioned previously, we use an equi-dimensional CG approach to provide target quantities for the total flux $Q_k\,$ defined in Equation (4.1), and the nodal distribution of the interface flux $\textbf{q}_k$ (with $k\in \{1,2\}$) defined in Equations (3.13)-(3.14). In that approach, fluid flow in fractured media is modeled as a standard diffusion problem with heterogeneous permeability, taking into account the differences in permeability between the surrounding background matrix and the fracture network. Specifically, the target solutions are calculated on a structured mesh with 4000 subdivisions in each direction in the background and 16 elements within the fracture thickness, and about $16\times 10^6$ dofs.

**Fig. 4:**
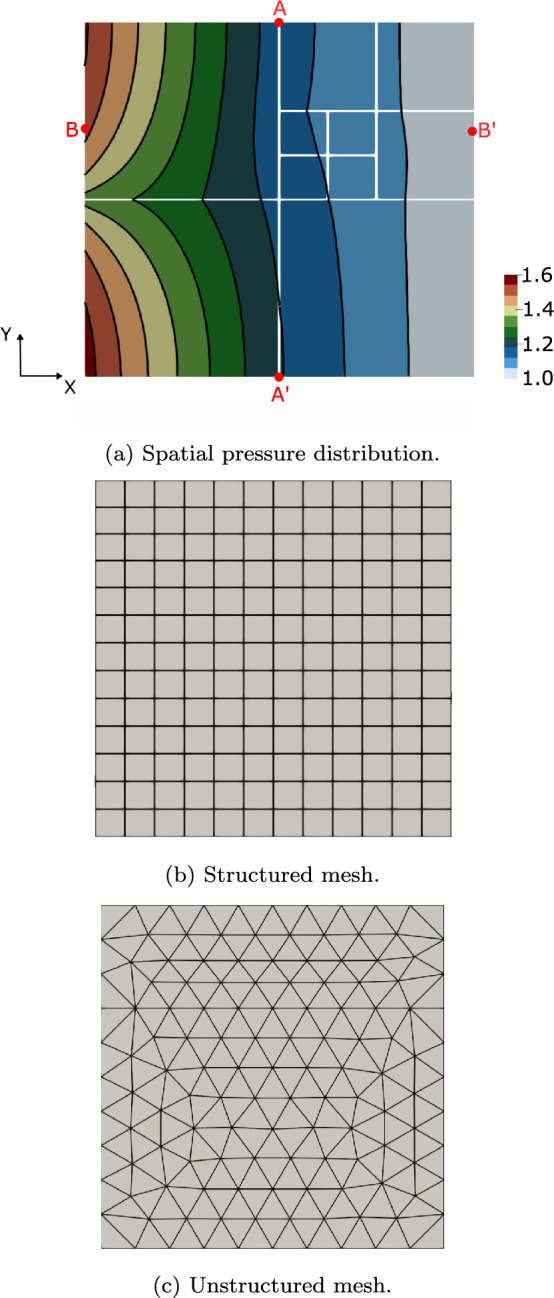
**(a)** Regular fracture network benchmark: spatial pressure distribution for the *unstructured* test case 3. We also report the extreme points of the two segments along which interface fluxes are computed. *Structured* **(b)** and *unstructured* **(c)** meshes for the background matrix of test case 0

Embedded-HD approach

We consider the same settings used in Flemisch et al. [11] where the fracture is represented as a one-dimensional object embedded in a two-dimensional square domain.

We use structured and unstructured mesh for the background matrix.

The characteristics of the meshes adopted for the *Embedded-HD* are reported in Table 3 for both the *structured* meshes, and for the *unstructured* meshes. Here, the name assigned to each test case refers to the number of uniform refinements.

Fluxes profiles are analyzed along the segments $\text {A}\text {A}^{\prime }$ ($x=0,5$ [m]) and $\text {B}\text {B}^{\prime }$ ($y=0,70$ [m]) as depicted in Figure 4a where we also depict the spatial distribution of the pressure for the test case 3. Figures 4b and 4c show the *structured* and *unstructured* meshes for the background matrix of test case 0.

**Table 4:** Regular fracture network benchmark: total fluxes for the *Embedded-HD* method. The total fluxes $Q_k$ with $k \in \{ 1,2\}$ are computed as defined in Equation (4.1) along segment $\text{ B }\text{ B}^{\prime }$

Mesh	Test case	$Q_1$	$Q_2$
	Target	0,117750	-0,117750
*Structured*	0	0,12015	-0,12015
	1	0,118149	-0,118149
	2	0,117903	-0,117903
	3	0,117852	-0,117852
	4	0,117812	-0,117812
	5	0,117792	-0,117792
	6	0,117765	-0,117765
	**7**	**0,117751**	**-0,117751**
*Unstructured*	0	0,119004	-0,119004
	1	0,118149	-0,118149
	2	0,118989	-0,118989
	3	0,118299	-0,118299
	4	0,118044	-0,118044
	5	0,117954	-0,117954
	6	0,117788	-0,117788
	**7**	**0,117757**	**-0,117757**

In Table 4 we report the total fluxes $Q_k$ with $k \in \{ 1,2\}$ computed along the segment $\text {B}\text {B}^{\prime }$ and compare them against the target solution.

**Fig. 5:**
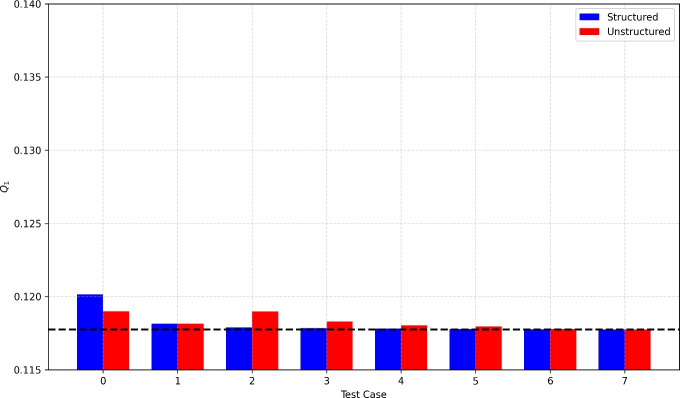
Regular fracture network benchmark: total flux $Q_1$ for the *Embedded-HD* method. The red and blue bars correspond to the flux computed for the structured and unstructured meshes, respectively. The black line represents the target flux. The labels along the *x*-axis refer to the mesh used for each test case

We observed that progressively increasing the resolution of the meshes–both for the background matrix and the fracture network–enables us to closely replicate the target solution. This trend is clearly illustrated in Figure 5 where we use a bar chart to depict the total fluxes for each test case.

As an additional test, we calculate the total fluxes along the segment, $\text {A}\text {A}^{\prime }$ where the exact solution is the Neumann boundary condition applied to the domain’s left side. For all numerical test cases, we recover the exact values $Q_1=1$ and $Q_2=-1$ (not shown in the Table).

**Fig. 6:**
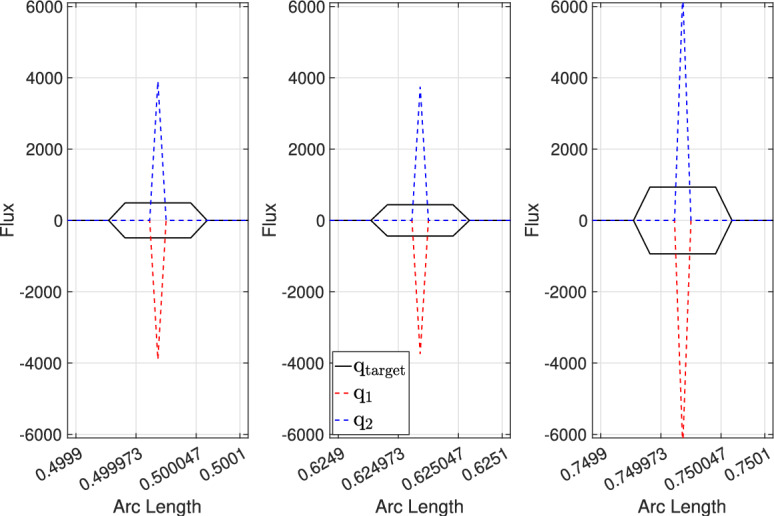
Regular fracture network benchmark: nodal distribution of the interface fluxes along $\text {B}\text {B}^{\prime }$. We compare the interface flux for the test case 2 against the target solution (black line)

It is worth noting that the *Embedded-HD* approach cannot fully capture the exact nodal distribution of the interface fluxes. Figure 6 shows the pointwise flux computed for the finest *Embedded-HD* case and compares it to the target distribution. The results confirm that the nodal flux distribution is conservative, but it approximates a Dirac delta distribution due to the lower dimensionality of the fracture network. Nevertheless, we emphasize that the total flux matches the target value.

This is the main reason we rely solely on the *Embedded-ED* approach to analyze point-wise flux.

Embedded-ED approach

We extend the analysis to the equi-dimensional embedded model, where the fracture network forms a two-dimensional domain within the same square background matrix. As before, we use both structured and unstructured meshes for the background matrix, while for the fracture network, we employ unstructured meshes.

**Table 5:** Regular fracture network benchmark: mesh characteristics for the *structured* and *unstructured* *Embedded-ED* test cases. We report the number of refinements in the second column for each test case, the number of elements and nodes for the meshes of the background matrix (**#E-matr.**, and **#N-matr.**) and of the fracture network (**#E-frac.**, and **#N-frac.**)

**Mesh**	Test case	#E-matr.	#N-matr.	#E-frac.	#N-frac.
*Structured*	0	1764	1 849	797	791
	1	6 889	7 056	909	903
	2	27 889	28 224	1181	1175
	3	62 500	63 001	1381	1375
	4	449 570	450 912	3065	3059
*Unstructured*	0	1 412	758	797	791
	1	5 582	2 942	909	903
	2	22 908	11 655	1181	1175
	3	172 872	87 025	1381	1375
	4	655 512	163 878	3 065	3 059

The characteristics of the meshes adopted for the *Embedded-ED* are reported in Table 5 for the *structured* and the *unstructured* meshes. Again, the name assigned to each test case refers to the number of uniform refinements performed in the background matrix.

**Fig. 7:**
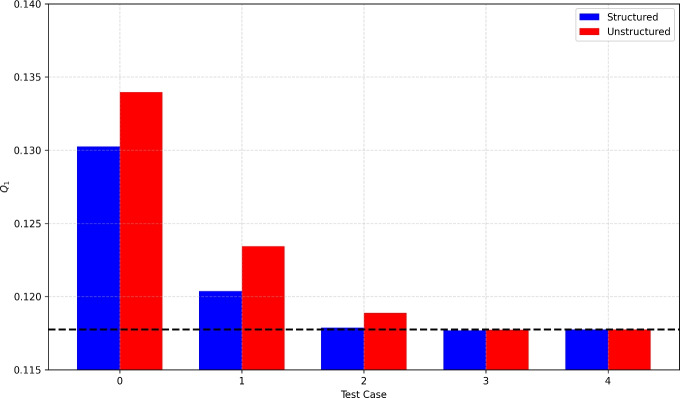
Regular fracture network benchmark: total flux $Q_1$ for the *Embedded-ED* method. The red and blue bars correspond to the flux computed for the structured and unstructured meshes, respectively. The black line represents the target flux. The labels along the *x*-axis refer to the mesh used for each test case

As already presented for the *Embedded-HD* approach, we compute the total fluxes along the segment $\text {A}\text {A}^{\prime }$ and find a perfect match with the Neumann boundary condition imposed on the left side of the domain for all the numerical test cases (not shown in Table 6). In Table 6, we report the value of the total fluxes computed along the segment $\text {B}\text {B}^{\prime }$. The results show that the flux is conservative and there is a very good match between the numerical fluxes computed for the finest test case and the target solution. Figure 7 presents the bar chart of the total fluxes for both structured and unstructured meshes, and compares them to the target flux. Once again, the results demonstrate that progressively increasing the mesh resolution allows for a closer match to the target solution.

**Table 6:** Regular fracture network benchmark: total fluxes for the *Embedded-ED* method. We compute the total fluxes $Q_k$ with $k \in \{ 1,2\}$ as defined in Equation (4.1) along the segment $\text{ B }\text{ B}^{\prime }$

Mesh	Test case	$Q_1$	$Q_2$
	Target	0,117750	-0,117750
*Structured*	0	0,130251	-0,130251
	1	0,120378	-0,120378
	2	0,117887	-0,117887
	3	0,117684	-0,117684
	**4**	**0,117751**	**-0,117751**
*Unstructured*	0	0,133958	-0,133958
	1	0,123439	-0,123439
	2	0,118882	-0,118882
	3	0,117739	-0,117739
	**4**	**0,117754**	**-0,117754**

The results analyzed so far demonstrate that embedded strategies are conservative for subdomains formed by the union of connected elements.

The second objective of this paper is to demonstrate that a point-wise identical balancing flux is recovered, ensuring uniqueness at the interface between the two subdomains. To this end, we analyze the nodal distribution of the interface fluxes $\textbf{q}_k$ defined in Equations (3.13)-(3.14) along the segment $\text {B}\text {B}^{\prime }$ and compare our numerical results with the target solution.

**Fig. 8:**
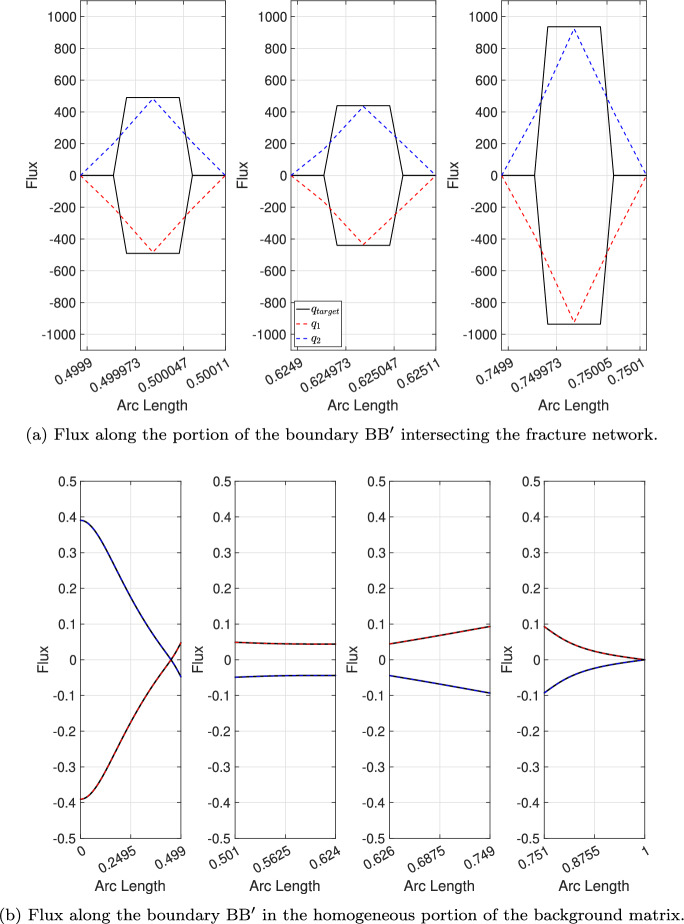
Regular fracture network benchmark: nodal distribution of the interface flux along the segment $\text {B}\text {B}^{\prime }$. We compare the interface flux for the test case 1 with the target solution (black line). In figure (a) from the left side to the right side we depict the flux distribution in the regions where the segment $\text {B}\text {B}^{\prime }$ overlaps with the fracture network, specifically around the points $x = 0.5$, $x = 0.625$, and $x = 0.75$

**Fig. 9:**
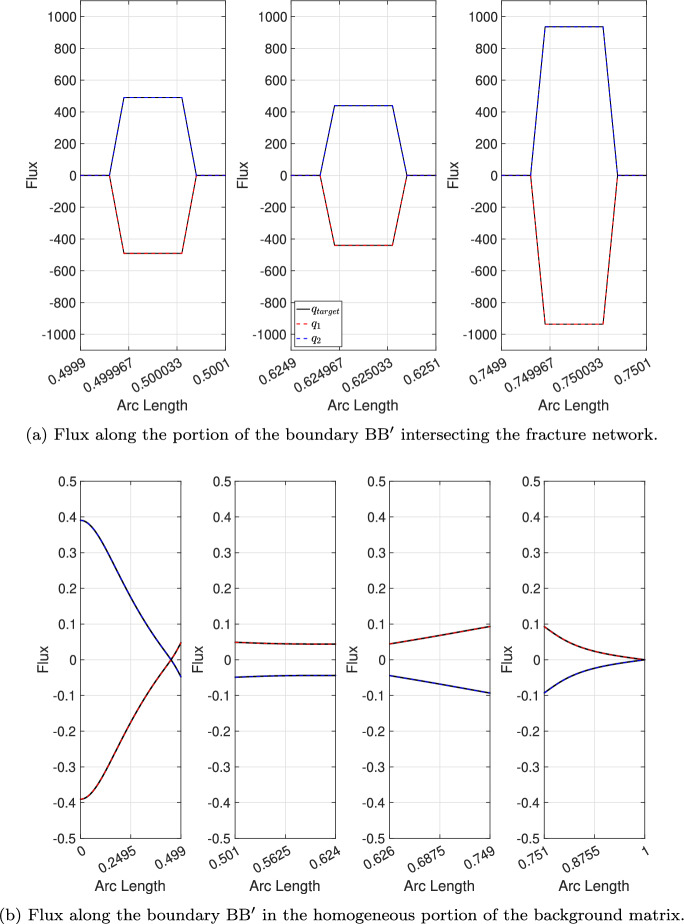
Regular fracture network benchmark: nodal distribution of the interface fluxes along $\text {B}\text {B}^{\prime }$. We compare the interface flux for the test case 2 against the target solution (black line). In figure (a) from the left side to the right side we depict the flux distribution in the regions where the segment $\text {B}\text {B}^{\prime }$ overlaps with the fracture network, specifically around the points $x = 0.5$, $x = 0.625$, and $x = 0.75$

In this analysis, we use structured meshes for the background matrix and provide their characteristics in Table 7. To accurately capture the nodal flux distribution, the background matrix mesh is refined in the regions overlapping the fracture network, particularly near the points $x = 0.5$, $x = 0.625$, and $x = 0.75$ (see Figure 4a ). Two test cases are examined, differing in the number of elements in the region of the background matrix mesh that overlaps with the fracture, as indicated in the sixth column of Table 7.

Figures 8 and 9 present a comparison of the nodal distribution of interface fluxes computed using the *Embedded-ED* method for both the coarser and finest test cases with the target solution. In these figures, the results computed using the *Embedded-ED* strategy are shown in blue and red, while the black line represents the target solution. We observe three distinct jumps at the intersections between the background matrix and the fracture network along the segment $\text {B}\text {B}^{\prime }$. Specifically, in Figures 8a and 8b, we focus on the overlapping region between the fracture network and the interface line $\text {B}\text {B}^{\prime }$, whereas Figures 8a and 8b show the remaining fluxes for the coarser and finest test cases, respectively.

**Table 7:** Regular fracture network benchmark: mesh characteristics for the *Structured Embedded-ED* test cases used in the analysis of nodal interface flux distribution. For each test case, we report the number of elements and nodes for the meshes of the background matrix (**#E-matr.** and **#N-matr.** ) and of the fracture network (**#E-frac.** and **#N-frac.**), the number of layers of elements (**#Layers**) in the background matrix mesh around the fracture network, and the deviation $e_q$ defined in Equation (4.2)

Test case	#E-matr.	#N-matr.	#E-frac.	#N-frac.	**#Layers**	$e_q$
1	8 464	8 742	1 360	1 685	2	1,8e-2
2	301 401	303 050	7 264	9 065	16	8,2e-4

In order to evaluate the accuracy of the nodal distribution of the interface fluxes $\textbf{q}_k$ with $k\in \{1,2\}$, we compute the deviation between our numerical results and the target solution based on the $L^2$ norm:

4.2 $$\begin{aligned} e_q^k=\Vert \textbf{q}_k-\textbf{q}_{k}^{R}\Vert _{L^2(\Omega _{m_k})}, \end{aligned}$$

where $\textbf{q}_{k}^R$ denotes the target solution for the interface flux. Given the uniqueness of the interface flux, we can omit the dependence on *k*.

The numerical results demonstrate the need for a sufficient number of elements around the fracture to accurately approximate the target solution. This is further highlighted by the deviations computed with respect to the target solution for the two test cases, as reported in the last column of Table 7. It is worth noting that the deviation, as defined in Equation (4.2), decreases with an increasing number of refinements and additional layers of elements in the region of the background matrix mesh that overlaps with the fracture network.

### Network with small features: material properties and geometrical settings

This benchmark involves a three-dimensional background matrix

$$ \Omega _m = (0\,\textrm{m}, 1\,\textrm{m}) \times (0\,\textrm{m}, 2.25\,\textrm{m}) \times (0\,\textrm{m}, 1\,\textrm{m}), $$

which contains a lower dimensional fracture network consisting of eight fractures as described in [2].

The boundary conditions for the background matrix are defined as follows. The Dirichlet boundary is given by

$$ \Gamma _m^D = \Gamma _{\text {out},0} \cup \Gamma _{\text {out},1}, $$

with

$$ \Gamma _{\text {out},0} = (0,1) \times \{2.25\} \times \left( 0, \tfrac{1}{3} \right) , \quad \Gamma _{\text {out},1} = (0,1) \times \{2.25\} \times \left( \tfrac{2}{3}, 1 \right) . $$

and the non-homogeneous Neumann boundary is defined as

$$ \Gamma _m^N = (0,1) \times \{0\} \times \left( \tfrac{1}{3}, \tfrac{2}{3}\right) . $$

We apply homogeneous Dirichlet conditions on $\Gamma _m^D$, and prescribe a uniform unit inflow on the inflow boundary $\Gamma _m^N$, such that

$$ \int _{\Gamma _m^N} \varvec{K}_m\nabla \varvec{p}_m \cdot \varvec{n} \, \textrm{d}S = -\frac{1}{3} \; [\textrm{m}^3/\textrm{s}]. $$

Homogeneous Neumann conditions are applied on the remaining boundaries.

The fractures are lower-dimensional entities, defined by their vertices as described in [2]. Material properties are listed in Table 8.

**Fig. 10:**
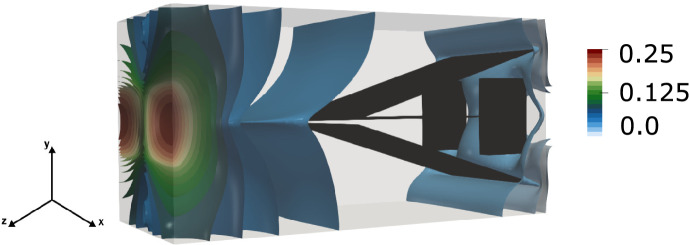
Network with small features benchmark: spatial pressure distribution

The computational mesh for the background matrix consists of $839\,808$ elements and $839\,808$ nodes. The mesh for the fracture network contains $7\,839$ elements and $4\,177$ nodes. A detailed numerical validation of this benchmark is presented in [2]. Figure 10 presents the spatial distribution of the pressure in the background matrix.

**Table 8:** Network with small features benchmark: material properties

Property	Symbol	Value	Unit
Fracture aperture	$\epsilon$	$10^{-2}$	m
Background matrix permeability	$k_m$	1	m/s
Fracture network permeability	$k_f$	$10^2$	m/s

To verify the local conservation, we compute the total flux across the plane $y = 1.0$, obtaining $Q_1 = 0.33333$ and $Q_2 = - 0.33333$. These values exactly match the analytical solution derived from the Neumann boundary condition imposed on the left side of the domain.

This result confirms that the numerical method conserves mass across internal interfaces, even in the presence of complex configurations and small-scale geometric features designed to challenge the method’s robustness and accuracy.

## Conclusions

In this study, we extended the conservation results for CG discretizations presented in  [17] to embedded strategies based on non-conforming meshes and dual Lagrange multipliers. Our numerical experiments demonstrate that the embedded methods presented in [45] exhibt local conservation properties. Both the hybrid- and equi-dimensional models of our approach effectively produce matching fluxes at the interface. The numerical results highlight the robustness of the conservation principle, even under relatively coarse mesh resolutions or in the presence of modeling errors associated with embedded hybrid-dimensional models. This robustness was also confirmed in three-dimensional scenarios, further validating the method’s applicability in complex geometrie. While the method guarantees conservation regardless of the mesh resolution, accurately evaluating the nodal boundary flux for equi- dimensional models may require adaptive mesh refinement, especially to capture jumps across fracture networks, as evidenced by the presented error analysis.

## Appendices

In this Appendix, we provide more details on the derivation of the nodal fluxes reported in subsection 3.3.

Equations (3.9) and (3.11), and Equations (3.10) and (3.12) are coupled with the continuity conditions:

$$\begin{aligned} \textbf{D}\textbf{p}_{f}=\textbf{B}\textbf{p}_m. \end{aligned}$$

It follows that:

6.1 $$\begin{aligned} \textbf{p}_{f}=\textbf{D}^{-1}\textbf{B}\textbf{p}_m. \end{aligned}$$

By substituting Equation (6.1) in Equations (3.11) and (3.12) one may get

6.2 $$\begin{aligned} \textbf{M}_l\textbf{q}(\Omega _{f_1})= \textbf{A}_{f_1}\textbf{D}^{-1}\textbf{B}\textbf{p}_m + \textbf{D}_{1}^T \varvec{\lambda } -\textbf{M}_f^D\textbf{q}_f^D, \end{aligned}$$

and

6.3 $$\begin{aligned} \textbf{M}_l\textbf{q}(\Omega _{f_2})= \textbf{A}_{f_2}\textbf{D}^{-1}\textbf{B}\textbf{p}_m + \textbf{D}_{2}^T \varvec{\lambda }. \end{aligned}$$

We can compute the value of $\varvec{\lambda }$ from Equations (6.2) and (6.3) and substitute it into Equations (3.9) and  (3.10). Thus, we get:

$$\begin{aligned} \textbf{M}_L\textbf{q}(\Omega _{m_1}) = \textbf{A}_{m_1}\textbf{p}_m + \textbf{T}_1^T \textbf{A}_{f_1} \textbf{T} \textbf{p}_m - \textbf{M}_m^D\textbf{q}_m^D-\textbf{T}_1^T\textbf{M}_f^D\textbf{q}_f^D -\textbf{T}_1^T\textbf{M}_l\textbf{q}(\Omega _{f_1}) , \end{aligned}$$

and

$$\begin{aligned} \textbf{M}_L\textbf{q}(\Omega _{m_2})= \textbf{A}_{m_2}\textbf{p}_m + \textbf{T}_2^T \textbf{A}_{f_2} \textbf{T}\textbf{p}_m - \textbf{f}_m -\textbf{T}_2^T\textbf{M}_l\textbf{q}(\Omega _{f_2}) , \end{aligned}$$

where $\textbf{T}_k=\textbf{D}_k^{-1}\textbf{B}_k$, $\textbf{T}=\textbf{D}^{-1}\textbf{B}$, and $\textbf{q}(\Omega _{k})$ is a coefficient vector of the total boundary flux. It follows that:

$$\begin{aligned} \textbf{M}_L\textbf{q}(\Omega _{m_1}) + \textbf{T}_1^T\textbf{M}_l\textbf{q}(\Omega _{f_1})&= \textbf{A}_{m_1}\textbf{p}_m + \textbf{T}_1^T \textbf{A}_{f_1} \textbf{T} \textbf{p}_m - \textbf{M}_m^D\textbf{q}_m^D-\textbf{T}_1^T\textbf{M}_f^D\textbf{q}^D_f, \\ \textbf{M}_L\textbf{q}(\Omega _{m_2}) +\textbf{T}_2^T\textbf{M}_l\textbf{q}(\Omega _{f_2})&= \textbf{A}_{m_2}\textbf{p}_m + \textbf{T}_2^T \textbf{A}_{f_2} \textbf{T}\textbf{p}_m - \textbf{f}_m, \end{aligned}$$

which recover Equations (3.13) and (3.14).
